# *Spondias pinnata* bark extract- an ameliorator of inflammatory derangement in etoposide induced mucositis: An experimental approach

**DOI:** 10.14202/vetworld.2021.1822-1828

**Published:** 2021-07-15

**Authors:** Aradhana Marathe, Gayathri M. Rao, M. Chakrapani

**Affiliations:** 1 Department of Biochemistry, Kasturba Medical College, Mangalore, Manipal Academy of Higher Education, Manipal, Karnataka, India; 2 Department of Medicine, Kasturba Medical College, Mangalore, Manipal Academy of Higher Education, Manipal, Karnataka, India.

**Keywords:** etoposide, mucositis, oxidative and inflammatory markers

## Abstract

**Background and Aim:**

Mucositis, one of the vulnerabilities of chemotherapy, affects the physiological activities and therapeutic strategies of patients because it can affect the normal cell population. Etoposide is a commonly used chemotherapeutic agent for cancers such as oral, lung, and gastrointestinal. In addition to the abnormal metabolic processes in the body caused by tumorigenesis, new metabolic alterations can occur, such as oxidative stress, antioxidant imbalance, and inflammatory reactions, all of which can contribute to existing patient vulnerability. Therapeutic adjuvants can help overcome these toxic effects. *Spondias pinnata* is a tropical tree omnipresent in the coastal and Western Ghat section of India that is used for culinary purposes and as a local analgesic. Therefore, we aimed to study the anti-inflammatory effects of *S. pinnata* in an etoposide-induced mucositis rat model.

**Materials and Methods:**

Small intestinal tissue homogenates from albino Wistar rats were used to estimate the levels of glutathione (GSH) and nitric oxide (NO), and activities of total antioxidant (TAO), myeloperoxidase (MPO) and Na^+^-K^+^ ATPase. The animals were grouped into: (1) normal control, (2) etoposide-induced mucositis (65 mg/kg bodyweight, single *IP* dose), (3) *S. pinnata* control group, and (4) etoposide followed by *S. pinnata* bark extract (200 mg/kg bodyweight, once in a day). Animals were sacrificed after 24, 48, 72, and 96 h and compared with that of the normal control group (n=6). Statistical analysis was performed using EZR software.

**Results:**

We observed a significant decrease in the TAO and GSH levels with a marked increase in NO, MPO, and Na^+^-K^+^ ATPase activity in the mucositis group. A tendency to recover from the decreased TAO and GSH levels existed in the treated group, showing the protective effects of *S. pinnata* bark extract against mucositis. In addition, this extract also showed anti-inflammatory effects as reflected by the recovery in MPO levels at the end of 96 h. Maintenance of Na^+^-K^+^ ATPase activity in the treated group demonstrates the protective effects of the extract against the increased levels observed in the etoposide-induced mucositis group.

**Conclusion:**

This study revealed the protective effects of *S. pinnata* bark extract against the oxidative and inflammatory changes that occurred during the development ofmucositis. This would decrease the pathological burden during chemotherapy and prevent any hurdles in therapeutic modalities.

## Introduction

Chemotherapy is the most common therapeutic strategy used worldwide during cancer treatment. Although this therapy is most promising, it attacks several normal body processes, worsening patients’ physiological vulnerability. Mucositis is one such outcome [1]. Etoposide, as an epipodophyllotoxin derivative and a topoisomerase-II inhibitor, is widely used in the treatment of various cancers [2].

Almost all chemotherapeutic agents cause mucotoxic effects that can make the life of cancer patients more miserable. Mucositis is the painful ulceration of the mucosal lining right from the oral cavity distributed across the gastrointestinal tract (GIT), affecting its normal functioning. Sonis [3] has explicitly divided the sequence of mucositis into five stages: Initiation, primary damage response, signal amplification, ulceration, and healing.

The pathophysiology of mucositis is still obscure; its occurrence lasts for about 3 weeks; and are of two types: direct and indirect. Chemotherapy interferes with the normal turnover of intestinal epithelial cells through one or both of these processes [4,5]. Generation of reactive oxygen species (ROS) happens during the initiation phase which, in turn, elicits the inflammatory reactions leading to the ablation of the epithelium and ulceration of the GIT, causing infiltration of microbiota. In the final phase, although healing occurs, the magnitude of the recovery depends on the extent of damage caused to the epithelium of the GIT, as well as the available defensive mechanisms [6,7]. Thus, an inflammatory cascade is exacerbated by the oxidative damage caused during mucositis.

Nitric oxide (NO), a principal inflammatory marker, is synthesized by inducible NO synthase (iNOS) during inflammation. Myeloperoxidase (MPO), the activity of which increases during systemic inflammation, also has been found to upregulate the activity of iNOS [8]. Reduced glutathione (GSH) and total antioxidant (TAO) activity are basic parameters that can help explain antioxidant status. Diarrhea is a common complication associated with mucositis, which alters the osmotic and ionic balance in the gut. Na^+^-K^+^ ATPase, an active enzyme of the intestinal epithelium, is required for the maintenance of the electrolyte balance attributed to its increased activity during chronic and acute inflammatory conditions of the GIT as well as is a signal transducer for muscle and nerve conduction [9,10]. Na^+^-K^+^ ATPase, which results in the production of pro-inflammatory cytokines and various other chemokines such as tumor necrosis factor-a, interleukin-5, nuclear factor-kB through mitogen-activated protein kinase, and p38 associated pathways [11,12], is now being treated as a target parameter in anticancer therapy.

Chemotherapy not only can pose a socioeconomic burden on patients but also can lead to psychological encumbrance due to the inexhaustible metabolic changes and side effects. Therefore, considering the infinitesimal molecular changes in an individual are necessary. In addition, several oxidative fluctuations followed by inflammatory archetypes can differ from one patient to another. The use of chemotherapeutic regimens, with their diverse combinations, action mechanisms, exposure, and patient capacity to withstand these, has become a crucial area of concern [13,14].

*Spondias pinnata*, also called the hog plum, is a deciduous tree belonging to the *Anacardaciae* family. The tree is found along the Western Ghats and the coastal belt of India and is used as a culinary and medicinal supplement. Methanolic and ethanolic bark extract studies with this plant have revealed the antibacterial, anticancer, anti-helminthic, analgesic, and antipyretic properties attributed to the presence of polyphenolic compounds [15].

Many naturally occurring herbs and herbal products have been used to relieve the severity of mucositis caused by chemotherapy. With this basic knowledge, we intended to study the protective effect of *S. pinnata* for its anti-inflammatory effect if any.

## Materials and Methods

### Ethical approval

Institution’s Animal Ethics Committee approved the study with EC Number: KMC/MNG/IAEC/05-2018),

### Study period and location

The study was conducted from June 2020 to December 2020 at Department of Biochemistry, Kasturba Medical College, Mangalore.

### Preparation of the *S. pinnata* bark extract

*S. pinnata*, bark was collected locally from Dakshina Kannada, and was identified and authenticated by Prof. Vasundhara Rao (retired professor), Department of Botany, Govt. College, Mangalore University and an ayurvedic physician.

We separated, shade-dried, and powdered the bark in a grinder. We extracted 100 g of the powder repeatedly with methanol (30%) using the Soxhlet apparatus over 72 h. We collected the concentrated bark extract and removed the solvent using a rotary evaporator. This semisolid extract was lyophilized and stored atroom temperature (27-30ºC) until use.

### Animals and groupings

We procured 78 rats from the central animal house, Kasturba Medical College, Mangalore, India, and housed these in polypropylene cages with husk as bedding material and standard food and water *ad libitum*. We divided the rats into four groups, subdividing each into a, b, c, and d subgroup related to the time of sacrifice, as follows:

- Group-1: Normal control group;
- Group-2: Mucositis model (EP): Subdivided into 2a, 2b, 2c, and 2d;
- Group-3: *S. pinnata* control group (SP): Subdivided into 3a, 3b, 3c, and 3d; and
- Group-4: *S. pinnata* and mucositis treatment group (EP+SP): Subdivided into 4a, 4b, 4c, and 4d.
- We considered the time of etoposide injection as 0 h and then calculated the time of exposure.

For Group 1, rats without any treatment and housed at normal temperature, humidity, and normal dietary conditions were considered as normal controls. For Group 2, mucositis was developed by etoposide administration in a single dose (65 mg/kg bodyweight) by intraperitoneal injection. Group 3 received 200 mg/kg bodyweight of *S. pinnata* bark extract once a day at 0, 24, 48, and 72 h. Group 4 was treated with *S. pinnata* once a day at 0, 24, 48, and 72 h, after EP administration and was considered as the treatment group. Animals were sacrificed at 24, 48, 72, and 96 h after intervention by euthanasia followed by cervical dislocation.

### Biochemical estimations

After sacrifice, the intestine was dissected, and 10% tissue homogenate was preparedusing phosphate-buffered saline, except for Na^+^-K^+^ ATPase, for which the tissue homogenate was prepared using a sucrose buffer.

1. Estimation of Na^+^-K^+^ ATPase activity: The method adopted was that by Adam-Vizi and Seregi [16] in which activity was calculated by the difference between the two assays. One unit of enzyme activity is expressed as U/L.
2. Estimation of MPO activity: The method adopted was described bySuchitra *et al*. [17] the enzyme activity is expressed as U/L.
3. Estimation of NO levels: The method adopted was described byMenaka *et al*. [18]. The level of NO is expressed as mg/g of tissue.
4. Estimation of reduced GSH: The method adopted was described by Ellman using DTNB. The values are expressed as mg/g of tissue [19].
5. Estimation of TAO: The method adopted was described by Korecevic *et al*. [20] activity is expressed as mM/L.

### Statistical analysis

Normal data are expressed as mean±standard error of the mean. Non-normal data are expressed as the median with a 95% confidence interval. We performed the statistical analysis usingEZR statistical software, version 1.54. (Jichi Medical University Saitama Medical Centre, Tokyo, Japan). We used one-way analysis of variance and the Kruskal–Wallis test for multiple comparisons and the Mann–Whitney U- and sample *t-*test to find the differences between groups, considering p<0.05 as statistically significant.

## Results

### MPO activity

We observed a highly significant increase in MPO activity in the rats exposed to etoposide (Groups-2a, 2b, 2c, and 2d). MPO activity remained in the normal levels in the SP control groups (Groups-3a, 3b, 3c, and 3d), and recovery occurred in the EP+SP groups (Groups-4a, 4b, 4c, and 4d; Table-1).

**Table-1:** MPO activity.

Groups	MPO activity (U/L) (Mean±SEM)	p-value between individual groups and normal group by independent sample t-test	p-value between EP, SP, and EP+SP groups, respectively, by ANOVA
	
Normal (1)	56.67±6.97		
EP 24 h (2a)	53.22±9.01	0.767	<0.001*
EP 48 h (2b)	89.27±7.38	0.00861*	
EP 72 h (2c)	91.71±6.04	0.00486*	
EP 96 h (2d)	97.19±4.38	<0.001*	
SP 24 h (3a)	54.13±1.04	0.727	0.0017*
SP 48 h (3b)	53.02±0.51	0.614	
SP 72 h (3c)	41.01±1.79	0.055	
SP 96 h (3d)	39.48±0.79	0.0344*	
EP±SP 24 h (4a)	68.44±2.18	0.138	0.255
EP±SP 48 h (4b)	66.26±3.88	0.258	
EP±SP 72 h (4c)	62.46±2.54	0.453	
EP ±SP 96 h (4d)	58.05±3.33	0.872	
Comparison between normal and 24 h after treatment groups			0.161
Comparison between normal and 48 h after treatment groups			<0.001*
Comparison between normal and 72 h after treatment groups			<0.001*
Comparison between normal and 96 h after treatment groups			<0.001*

MPO=Myeloperoxidase, SEM=Standard error of the mean, ANOVA=Analysis of variance

### NO levels

A significant increase occurred in the EP groups over a period of 24-96 h. We observed an insignificant decrease in NO levels among the SP control groups over 24-96 h when compared with that of the normal control group. We also observed a significant decrease in NO levels in the EP+SP groups over 24-96 h. However, the levels remained high in the EP+SP groups when compared with the SP and normal control groups. Surprisingly, the results showed that the NO levels remained higher in EP+SP 24 h and EP+SP 48 h than the EP 24 h and EP 48 h levels respectively (Table-2).

**Table-2:** Nitric oxide levels.

Groups	NO concentration (mg/g tissue) (Mean±SEM)	p-value between individual groups and normal group by independent sample t-test	p-value between EP, SP, and EP+SP groups, respectively, by ANOVA
	
Normal (1)	14.35±0.77		
EP 24 h (2a)	18.42±0.64	0.00597*	<0.001*
EP 48 h (2b)	28.33±0.33	<0.001*	
EP 72 h (2c)	67.87±3.57	<0.001*	
EP 96 h (2d)	98.82±2.19	<0.001*	
SP 24 h (3a)	12.51±1.33	0.26	0.396
SP 48 h (3b)	12.37±0.54	0.0638	
SP 72 h (3c)	11.295±0.53	0.00878*	
SP 96 h (3d)	11.205±0.35	0.0042*	
EP±SP 24 h (4a)	70.02±3.9	<0.001*	<0.001*
EP±SP 48 h (4b)	57.10±5.63	<0.001*	
EP±SP 72 h (4c)	53.37±4.90	<0.001*	
EP±SP 96 h (4d)	47.736±2.72	<0.001*	
Comparison between normal and 24 h after treatment groups			<0.001*
Comparison between normal and 48 h after treatment groups			<0.001*
Comparison between normal and 72 h after treatment groups			<0.001*
Comparison between normal and 96 h after treatment groups			<0.001*

NO=Nitric oxide, SEM=Standard error of the mean, ANOVA=Analysis of variance

### TAO

A significant decrease in TAO activity occurred among the EP groups over 24-96 h. We observed non-significant difference among the SP control groups sacrificed at different intervals and a significant increase in TAO activity in the EP+SP groups over 24-96 h, which showed a tendency to return to the normal level at 96 h. (Table-3).

**Table-3:** TAO activity.

Groups	TAO activity (mM/L) (Mean±SEM)	p-value between individual groups and normal group by independent sample t-test	p-value between EP, SP, and EP+SP groups, respectively, by ANOVA
	
Normal (1)	8.91±0.13		
EP 24 h (2a)	8.0±0.2	0.00463*	<0.001*
EP 48 h (2b)	6.52±0.16	<0.001*	
EP 72 h (2c)	4.58±0.08	<0.001*	
EP 96 h (2d)	2.72±0.31	<0.001*	
SP 24 h (3a)	8.48±0.14	0.0518	0.0272*
SP 48 h (3b)	8.96±0.09	0.771	
SP 72 h (3c)	8.98±0.10	0.708	
SP 96 h (3d)	9.01±0.08	0.708	
EP±SP 24 h (4a)	6.73±0.2	<0.001*	<0.001*
EP±SP 48 h (4b)	7.55±0.14	<0.001*	
EP±SP 72 h (4c)	8.36±0.10	0.00885*	
EP±SP 96 h (4d)	8.60±0.04	0.0794	
Comparison between normal and 24 h after treatment groups			<0.001*
Comparison between normal and 48 h after treatment groups			<0.001*
Comparison between normal and 72 h after treatment groups			<0.001*
Comparison between normal and 96 h after treatment groups			<0.001*

TAO=Total antioxidant, SEM=Standard error of the mean, ANOVA=Analysis of variance

### GSH levels

A statistically significant, gradual decrease in GSH levels occurred in the EP groups, a significant increase in GSH levels occurred in the SP control groups over 24-96 h, and a gradual increase in GSH levels occurred in the EP+SP groups, with progress over the time. The levels remained significantly low in the EP+SP 24 h group in comparison with that of the normal control group; however, the levels had a tendency to increase toward normal (Table-4).

**Table-4:** GSH levels.

Groups	GSH levels (mg/g tissue) (Mean±SEM)	p-value (in comparison with normal group) by independent sample t-test	p-value between EP, SP, and EP+SP groups, respectively, obtained by ANOVA
	
Normal (1)	206.54±5.64		
EP 24 h (2a)	169.66±2.98	0.001*	<0.001*
EP 48 h (2b)	148.16±4.72	<0.001*	
EP 72 h (2c)	133.24±7.03	<0.001*	
EP 96 h (2d)	105.90±11.49	<0.001*	
SP 24 h (3a)	195.35±3.31	0.118	<0.001*
SP 48 h (3b)	200.95±5.18	0.482	
SP 72 h (3c)	216.69±0.99	0.107	
SP 96 h (3d)	222.70±2.82	0.0284*	
EP±SP 24 h (4a)	109.59±4.02	<0.001*	<0.001*
EP±SP 48 h (4b)	132.17±1.88	<0.001*	
EP±SP 72 h (4c)	142.53±1.77	<0.001*	
EP ±SP 96 h (4d)	159.60±2.62	<0.001*	
Comparison between normal and 24 h after treatment groups			<0.001*
Comparison between normal and 48 h after treatment groups			<0.001*
Comparison between normal and 72 h after treatment groups			<0.001*
Comparison between normal and 96 h after treatment groups			<0.001*

GSH=Glutathione, SEM=Standard error of the mean, ANOVA=Analysis of variance

### Na^+^-K^+^ ATPase activity

We observed a more than 50% decrease in the enzyme activity after 24 h, followed by a sudden increase in the activity after 48 h, with gradual hike at 72 and 96 h in the EP groups as compared with the other groups (Table-5).

**Table-5:** Na^+^-K^+^ ATPase activity.

Groups	Na^+^-K^+^ ATPase activity (U/L) Median (Q1, Q2)	p-value between individual groups and normal group obtained by Mann–Whitney U-test	p-value between EP, SP, and EP+SP groups, respectively, obtained by Kruskal–Wallis rank-sum test
	
Normal (1)	0.36 (0.22, 0.39)		
EP 24 h	0.16(0.07,0.27)	0.352	0.05*
EP 48 h	0.47 (0.23,1.31)	0.36	
EP 72 h	0.50 (0.34, 0.53)	0.329	
EP 96 h	0.67 (0.60,0.88)	0.00952*	
SP 24 h	0.25 (0.22,0.27)	0.336	0.3215
SP 48 h	0.27 (0.24, 0.28)	0.378	
SP 72 h	0.32 (0.30,0.33)	0.394	
SP 96 h	0.32(0.25,0.36)	0.873	
EP±SP 24 h	0.25 (0.23, 0.27)	0.429	0.0275*
EP±SP 48 h	0.26 (.24,0.27)	0.394	
EP±SP 72 h	0.28 (0.26,0.30)	0.378	
EP±SP 96 h	0.36 (0.34,0.38)	0.927	
Comparison between normal and 24 h after treatment groups			0.5445
Comparison between normal and 48 h after treatment groups			0.651
Comparison between normal and 72 h after treatment groups			0.197
Comparison between normal and 96 h after treatment groups			0.0197*

## Discussion

Cytotoxicity is a major side effect of chemotherapy. Inflammation and mucosal tissue injury are attributed to cytotoxicity. In addition, etoposide induces a severe form of mucositis, during which ROS generation triggered by etoposide leads to an inflated inflammatory response [3,4,21]. Redox homeostasis decides the effect of ROS on macromolecules, including nucleic acids, proteins, and enzymes.

DNA damage is a key process that affects the cell cycle at different levels. The changes during the development of mucositis highlight ROS-sensitive sequences, which elicit the inflammatory mechanisms. ROS stimulates the assembly of leucocytes in the damaged tissue, actuating neutrophil production. Activated neutrophils escalate the production of inflammatory marker MPO, a hemoprotein stored in the leukocyte granules. This increase is the basis for the inflammation due to ROS [22].

In the present study, we found an insignificant initial decrease in MPO activity at 24 h after etoposide exposure, which looks accidental and yet interesting. However, a sharp increase at 48 h and a subsequent increase thereafter can be regarded as aggravated mucositis. MPO catalyzes oxidation of the phenolic ring of etoposide to yield phenoxyl radical as shown below [23].

Etoposide-OH+RO**_2_** Etoposide--O**^•^**+RO**_2_**H

Etoposide--O**^•^**+RH Etoposide-OH +R**^•^**

The dip in the observed MPO levels could be caused by the time taken by etoposide metabolism, and an increase in the levels could be due to the enhanced radical production as a result of this metabolism. This mechanism increases the existing free radical pool to augment the propagating chain reaction that leads to the amplification of pro-inflammatory cytokines. Thus, formation of phenoxyl radicals from the etoposide parallels the increase in MPO activity at a later stage, as seen in our study. This finding also is corroborated by Atwal *et al*. [24] who have shown that MPO added to the etoposide-induced DNA damage through free radical toxicity.

We found a decrease in the MPO levels in the mucositis model treated with *S. pinnata* over 96 h, showing that this extract can be beneficial in treating the inflammation caused by chemotherapy. Catalytic activity of MPO also is influenced by NO in anaerobic conditions, and the increased activity of MPO decreases the bioavailability of NO for its normal functioning in the body [25]. This condition is evident in the present study.

In our study, we observed an increase in NO levels with the time of exposure to etoposide, emphasizing possible drug toxicity on the intestinal tissue to develop mucositis. Moreover, NO levels in the SP control groups corresponded to those of normal levels in rats and a decrease in the levels observed over 96 h. These results provide evidence of the protective action of *S. pinnata*, as the NO levels showed a tendency toward normal in the mucositis groups treated with the extract. NO, synthesized by the action of iNOS, is a short-living molecule that can easily diffuse through the tissues [26]. iNOS activity has been found to increase with the administration of chemotherapeutic regimens such as 5-fluorouracil in the esophageal cells [27]. At a higher concentration, NO reacting with superoxide radicals can release peroxynitrate radicals, which can aggravate tissue injury and inflammation through cytokine-mediated reactions [28].

Worsened antioxidant levels can occur in patients receiving chemotherapy [29]. In the present study, the rat intestinal tissue showed a significant gradual depletion of the antioxidant status due to etoposide treatment over 96 h, indicating oxidative constraint in the tissue along with the progress in time of exposure. In the SP control groups, we found an increase in antioxidant activity over 24-96 h, reflecting the antioxidant potential of *S. pinnata*. This finding is similar to that by Kumar, in which *S. pinnata* fruit showed high radical scavenging activity attributed to its phenolic and tannin content [30]. Our study also showed that treatment with this extract could maintain TAO at levels similar to those in normal control group, which could be due to the presence of the above-mentioned compounds that we observed during the component analysis, thus highlighting anti-mucositic effects. In addition, the analyzed GSH levels support this observation.

GSH shows a wide range of paradigms in cancer therapies [31,32]. In the current study, the intestinal GSH levels significantly decreased along with the increase in time after etoposide administration, indicating the loss of antioxidant potential in the rat intestinal tissue. We observed sustained GSH levels in the groups of rats exposed to etoposide and treated with *S. pinnata*. Our findings are supported by evidence from Iqbal *et al*. [33], in which *S. pinnata* bark extract attenuated the ethanol-induced hepatotoxicity, as well as by those from our previous study, reinforcing the maintained normal TAO levels of the same group [34].

Development of diarrhea is a key feature during mucositis, caused by the altered ionic status of the cellular environment. Maintenance of the chemical and electrical gradient between intra- and extra-cellular spaces is primarily done by Na^+^-K^+^ ATPase [35]. In our study, we observed a dip in the activity of intestinal Na^+^-K^+^ ATPase at 24 h after etoposide administration and also saw a parallel enhancement in inflammatory status. An interesting finding is an eventual rise in the level over the 96 h of administration, which could be attributed to the increased cellular destruction due to mucositis. Evidence from the literature also indicates that both the phosphorylation of amino acid residues and the localization of a-subunit of the enzyme can lead to its increased activity [36]. Nevertheless, treatment with *S. pinnata* along with etoposide showed that the conservation of the sodium pump activity can ensure the protective action of this bark extract against tissue injury and inflammation.

## Conclusion

Over the time, etoposide exposure causes oxidative stress-mediated mucosal tissue injury. The mechanisms discussed above, if monitored during chemotherapy, could be beneficial in curbing the general psychological and pathological burdens of the patient caused by mucositis and its consequences. Considering the basic needs of patients such as difficulty in consuming a proper diet, *S. pinnata* bark extract promises for minimizing the ill effects caused by chemotherapy and can help in planning further treatment strategies.

## Authors’ Contributions

GMR and MC: Research concept and design. GMR and AM: Collection and assembly of data, data analysis and interpretation, and writing the manuscript. MC: Expert advice for the concept and manuscript. All authors read, revised and approved the final manuscript.
